# Distinguishing fast change in social norms and slow change in personal norms in cooperative decision-making

**DOI:** 10.3389/fpsyg.2024.1380341

**Published:** 2024-05-30

**Authors:** Marlene C. L. Batzke, Andreas Ernst

**Affiliations:** Center for Environmental Systems Research, University of Kassel, Kassel, Germany

**Keywords:** social norms, personal norms, norm change, cooperation, prisoner’s dilemma game, decision-making

## Abstract

Psychological research on norms has shown that norms are highly relevant for individuals’ decision-making. Yet, there is so far little understanding of how norms change over time. Knowledge about how norms change may help better understanding their potential for as well as limitations in guiding decision-making and changing behavior. The present work investigated change in individuals’ cooperation norms. As an indicator of different underlying processes of norm change, the temporal dynamics of different types of norms were examined. It was assumed that participants’ social norms are adapted quickly whenever the social situation changes, while personal norms change more slowly and gradually, abstracting part of the situational learning in interaction with one’s personality. In an experimental study, participants played a repeated prisoner’s dilemma game with artificial co-players representing a predominantly cooperative or uncooperative social setting, depending on the experimental condition. The condition was expected to affect slow learning of personal norms. Additionally, the cooperativeness of the social setting was varied repeatedly within conditions, expected to result in fast changes in social norms. Participants’ personal and social norms were assessed throughout the game. As predicted, the temporal dynamics differed between norms with social norms changing quickly and personal norms more slowly. Personal norms strongly predicted behavioral decision-making and were predicted by situational and personality factors. Potential qualitative differences of the underlying norm change processes are discussed.

## 1 Introduction

Humans are social beings. We attend to what others do, what others believe, and form assumptions about what it is that others believe and do. We then use these assumptions to make accurate and appropriate behavioral decisions (Ajzen, 1991). One of the key features in how social influence affects behavior is described in the concept of social norms (Cialdini et al., 1990). Social norms describe what many people consider appropriate or normal behavior and they change over time – sometimes rapidly (as we have seen during the COVID-19 pandemic), and sometimes strikingly slowly. While individuals perceive and learn social norms, they also develop their own personal norms (Thøgersen, 2006). Personal norms may well differ from social and societal norms, being influenced by the individuals’ experiences, their social network, and so forth. Over the past decades, psychological research has well documented the power of social norms on individuals’ behavioral decision-making (Kallgren et al., 2000; Cialdini et al., 2006; Göckeritz et al., 2010). However, there is still little understanding of how norms dynamically unfold over time (van Kleef et al., 2019). How do norms develop and change? Knowledge about how norms change may help better understanding their potential for as well as limitations in guiding decision-making and changing behavior (Andrighetto and Vriens, 2022).

Norms can be defined as behavioral rules for a specific situation (Dannenberg et al., 2024). Social situations are informed by a multitude of social norms, being what many people consider appropriate or normal behavior. The power of social norms has long been known in psychology (Sherif, 1936; Deutsch and Gerard, 1955; Asch, 1956). People are highly sensitive to their social surrounding, yielding to social norm pressure and conforming to the group to gain social approval and avoid social sanctions (Cialdini et al., 1990; McDonald and Crandall, 2015; Nyborg et al., 2016). Providing people with information about social norms affects their behavior (Schultz et al., 2007, 2008; Goldstein et al., 2008; Keizer et al., 2008; Miller and Prentice, 2016) – even without them consciously knowing about it (e.g., Nolan et al., 2008). People tend to assimilate with social norms in the sense of imitation and conformism (Cialdini and Goldstein, 2004). Cialdini et al. (1990) demonstrated the distinct importance of two different qualities of social norms: the injunctive and descriptive quality. (Social) injunctive norms contain information about the (in)appropriateness of a behavior in a specific situation [i.e., what most others consider (in)appropriate], motivating through the promise of social (dis)approval. (Social) descriptive norms refer to the observable regularity/normality of a behavior in a specific situation (i.e., what most others do), motivating by “what will likely be effective and adaptive” (p. 1015).

Moreover, norms have been stated to function at different levels, meaning the social/societal and the individual level (Cialdini et al., 1990; Farrow et al., 2017; Bicchieri et al., 2018; Dannenberg et al., 2024). Hence, individuals not only perceive and tend to conform to social norms, but also develop their own personal norms, a concept most notably known by the work of Schwartz (1977; Schwartz and Howard, 1981, 1982). Personal norms can be defined as individuals’ beliefs about (in)appropriate behavior in a specific situation (Batzke and Ernst, 2023b). Thus, personal norms are of an injunctive quality. Research has shown that individuals’ personal norms strongly predict behavioral decisions (Hunecke et al., 2001; Klöckner and Matthies, 2004; Bamberg, 2013; Onwezen et al., 2013; Han, 2014; Szekely et al., 2021) and explain variance in behavioral decisions over and above social norms (Conner and Armitage, 1998; Harland et al., 1999; Shin et al., 2018). Although Steg and de Groot (2010) demonstrated that personal norms can be manipulated, there is little experimental evidence on the effects of personal norms, research being mostly survey based. Biel and Thøgersen (2007) stated that “there is a need for more research providing unambiguous research on that topic” (p. 105).

Apart from the quality and subject of a norm, norms can be differentiated by their orientation, being self-oriented (i.e., concerning the individual’s behavior) or other-oriented (i.e., concerning behavior of others). Social norms are most often implicitly conceptualized as other-oriented (e.g., what others do, rather than what others believe I do). Regarding personal norms, there is ambiguity in the literature. They have been operationalized in the sense of self-oriented personal norms [i.e., what I consider (in)appropriate for myself, see Hunecke et al., 2001; Klöckner and Matthies, 2004; Bamberg et al., 2007; Steg and De Groot, 2010; Bamberg, 2013; Han, 2014], as well as other-oriented personal norms [i.e., what I consider (in)appropriate for others, see Bicchieri et al., 2014] or a combination [i.e., what I consider (in)appropriate behavior in general, see Szekely et al., 2021; Bicchieri et al., 2022]. In the present work, self- and other-oriented social norms and personal norms are differentiated, which allows investigating potential differences. Hereafter, if not specified otherwise the term “personal norms” implies both types of orientations. Yet, the term “social norms” is used to refers only to other-oriented social (descriptive and injunctive) norms, if not specified otherwise.

Based on these different norm types, experimental norm research tends to focus on showing the causal effect of a type of norm on behavioral decisions (for reviews see Miller and Prentice, 2016; Bicchieri and Dimant, 2019). Behavioral adaptations are immediate (Cialdini and Goldstein, 2004; Nolan et al., 2008) and in direction of the presented social norm (e.g., Schultz et al., 2007). For instance, it was shown that participants, who were given the opportunity to litter, were more likely to do so in a littered environment than in a clean environment (Cialdini et al., 1990, Study 1). Yet, the topic of norm change in individuals has gained far less attention in experimental research (Andrighetto and Vriens, 2022). Theoretically, it is assumed that cognitive processes shape the individually learned norms, which in turn shape the social dynamics (Hawkins et al., 2019). Accordingly, acquiring norms is based on social cognition and social learning (Howard and Renfrow, 2003; Kelly and Davis, 2018; Theriault et al., 2021). It is further assumed that social constructs become internalized, meaning part of the individual’s identity or self, as described in self-determination theory (Ryan and Deci, 2000) or self-categorization theory (Turner, 1987). The idea of personal norm change can also be related to moral development theories, stating how children develop moral principles of right and wrong (Kohlberg, 1964; Piaget, 1970). Accordingly, it was assumed these principles are predominantly acquired in early childhood (Turiel, 1983; Nucci, 2001). However, taking a learning perspective on morality, ranging from intuitionist, emotional (e.g., Haidt, 2001) to rational, reward-maximizing approaches (e.g., Crockett, 2013; Cushman, 2013), it can also be assumed that any type of norm is acquired and may change throughout the lifetime (Chudek and Henrich, 2011; McDonald and Crandall, 2015; Kelly and Davis, 2018). Through repetition (Prentice and Paluck, 2020), social enforcement (Schultz et al., 2007), and internal feedback (Schwartz and Howard, 1981), new norms may develop and existing ones may change. So far, processes of norm change unfolding over time are little understood (Anderson and Dunning, 2014; Dannals and Miller, 2017; van Kleef et al., 2019; Dannals et al., 2022).

There are currently few approaches investigating norm change experimentally, especially when focusing on those that explicitly assessed participants’ social and personal norm change. In Szekely et al. (2021), participants were confronted with different collective risks in a long-term social dilemma experiment. It was shown that social norms are adapted along the within-subjects (high vs. low) risk variation. Effects on personal normative beliefs (i.e., personal norms) were not analyzed; descriptive data seemed inconclusive (see Szekely et al., 2021, Supplementary Figure S5). Similarly, Bicchieri et al. (2022) found that observing norm violations led participants to adapt their social norms, but not their personal normative beliefs. This might lead to believe that personal norms do in fact not change. Tverskoi et al. (2023) showed that they can change. Participants played an online common pool resources game for 35 days either with or without messaging. Personal norms as well as normative expectations and empirical expectations (relating to social injunctive and social descriptive norms) all became less cooperative over the course of the game (indicating higher resource extraction).

There are numerous approaches to studying norm change processes via modeling and simulation methods. While many of them conceptualize norms as social level phenomena of behavioral convergence (e.g., Axelrod, 1986; Sen and Airiau, 2007), fewer focused on norm change within the individual, which demands for representing norms as mental objects that artificial agents can deliberate upon (e.g., Dignum, 1999; Castelfranchi et al., 2000; Villatoro et al., 2015). Batzke and Ernst (2023b) introduced the idea of different norm learning processes possessing different temporal dynamics, meaning that they occur at different rates of change (see also Batzke and Ernst, 2022). Whereas social norm learning is presumably merely based on observation, it was assumed a fast adaptation process with a high rate of change. This relates to social norm research, showing immediate behavioral responses to social norm presentations (e.g., Cialdini and Goldstein, 2004; Schultz et al., 2007; Nolan et al., 2008). Personal norm learning, however, was assumed to be slower with a low rate of change, being influenced by situational (e.g., Hoffman, 2000) as well as personal factors (e.g., Ryan and Deci, 2017), abstracting part of the situational learning across situations. Personal norms were assumed to manifest subjective experiences of social norms and their individual evaluation over time. Hence, personal norm change was stated to be qualitatively differently, individually specific, and evolving over a longer period of time (i.e., to be slower).

In the present work, the assumption of temporal differences between social and personal norm processes was adopted, using the temporal dynamics as a proxy for potentially different underlying processes. Differentiating the temporal dynamics of social and personal norm change is a step toward understanding the mechanisms of norm change, which is “is crucial for identifying interventions which could lead to large-scale behavioral change” (Andrighetto and Vriens, 2022, p. 4) and fostering cooperative decision-making (Cushman et al., 2017). The purpose of the present work was to investigate social and personal norm change experimentally in a prisoner’s dilemma game. In a prisoner’s dilemma game, a player can choose each round between two actions: cooperation, being the collective beneficial action, and non-cooperation/defection, being the choice maximizing the own benefit. A player receives a higher payoff for the defecting choice; however, all players are better off if all cooperate rather than defect (Axelrod, 1984). Hence, the individual’s self-interest is at odds with the collective interest (Dawes, 1980). Repeated prisoner’s dilemmas existing of several rounds allow for the development of social norms (Peysakhovich and Rand, 2016). Norms have been shown to motivate cooperative behavior in prisoner’s dilemmas, influencing small group cooperation (Ostrom, 2000; Bicchieri et al., 2023) and creating tipping points for large-scale transformations (Nyborg et al., 2016; Otto et al., 2020).

**Figure 1:**
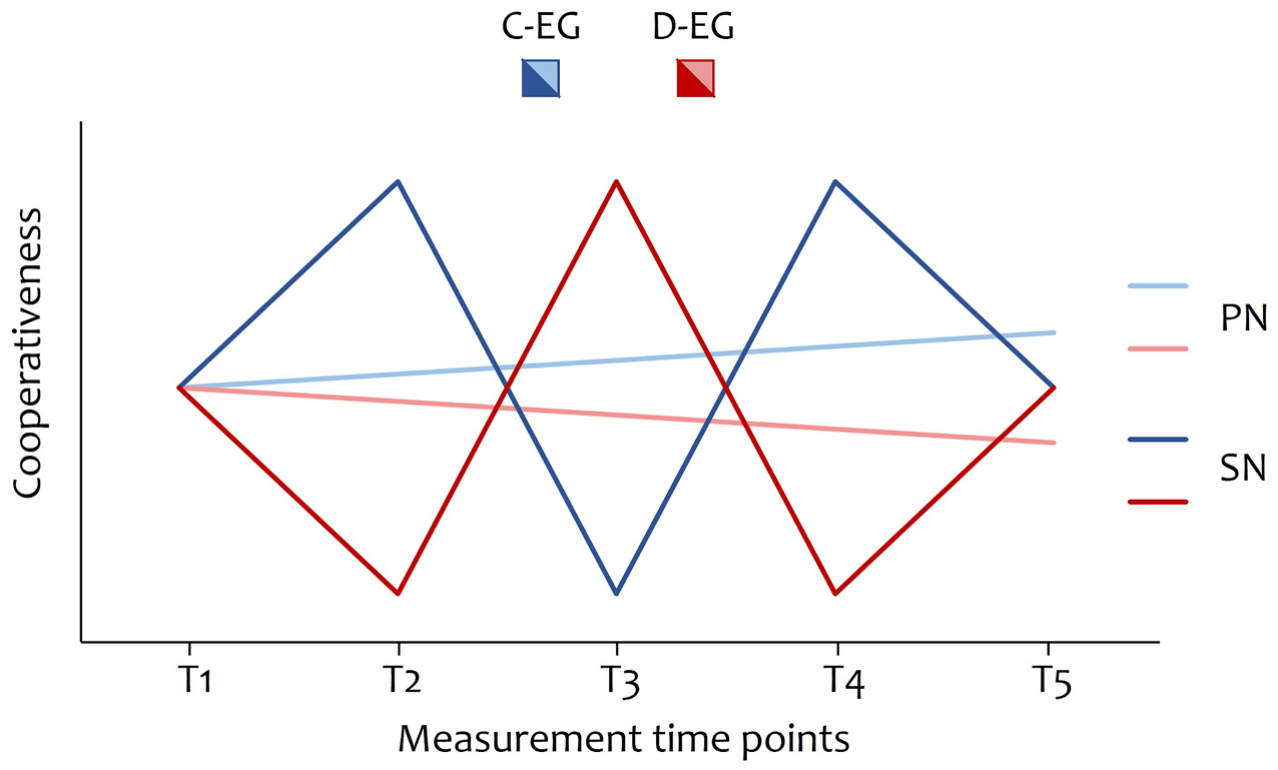
Hypothesized change in social and personal norms depending on the experimental group. C-EG, cooperative experimental group; D-EG, defective experimental group; PN, personal norms; SN, social norms.

To differentiate the temporal dynamics of participants’ social and personal norm change processes in a repeated prisoner’s dilemma game, participants were confronted with repeated changes in the cooperativeness of the social setting. It was assumed that participants would adapt their social norms quickly, according to the repeatedly changing cooperativeness of the social setting. Moreover, experimental groups differed in their overall cooperativeness. Participants’ personal norms were assumed to be adapted slowly according to the overall cooperativeness in the experimental group, which would show in group differences in personal norms after the game. The following research questions and corresponding hypotheses were addressed:

Do social and personal norms differ in their temporal dynamics, i.e., their rates of change?
  1.1. The average difference between single consecutive measurements is greater in social norms than in personal norms.
  1.2. Social norms are repeatedly reversed during the game in line with the cooperativeness of the social setting (in the sense of seasonal changes, see Figure 1).
  1.3. Personal norms change gradually during the game (in the sense of a linear trend, see Figure 1).
Can personal factors explain personal norm change over and above situational factors, while social norms are solely predicted by situational factors?
  2.1. Social norms are predicted by the experimental group.
  2.2. Personal norms are predicted by the experimental group, trait cooperativeness, and their interaction.
Do personal norms have an independent effect on behavioral decisions (over and above social norms)?
  3. In addition to social norms, personal norms explain variance in the follow-up behavior.
Can personal norms be influenced toward more cooperativeness?
  4.1. After the game, participants in the cooperative group show higher levels of personal norms of cooperation than those in the defective group.
  4.2. Participants in the cooperative group cooperate more in the follow-up behavior than those in the defective group.

## 2 Materials and methods

### 2.1 Sample

Using the online tool Glimmpse,^1^ a target sample size for obtaining a power of 0.95 at a 0.05 familywise alpha error probability for the interaction effect of the contrast analyses between time (within-subjects factor) and experimental group (between-subjects factor), formulated in Hypotheses 1.2 and 1.3, of *N* = 10 for social norms and *N* = 386 for personal norms was calculated (see preregistration for the specifics, https://osf.io/xgucf). In total, *N* = 440 participants were recruited in March/June 2022 and compensated via the survey institute Bilendi, assuming that some data had to be excluded according to the predefined exclusion criteria (see preregistration at https://osf.io/xgucf). Full-aged, German-speaking individuals were permitted to participate in the study. The sample was assessed according to age, gender, and income statistics for Germany.

From the initial pool, two participants were excluded due to being underage and eight participants due to low proficiency in the German language. Another 31 participants were excluded for pausing during the prisoner’s dilemma game for more than 3 min. Participants were directly instructed not to take breaks during the game, as breaks potentially exposed the cover story of participants playing an online game with real other participants. Three participants were excluded due to highly conspicuous item response patterns. Thirty-one participants were removed due to perceiving the game as extremely unreal (being statistical outliers) or, in the open questions at the end of the study, expressing serious doubts concerning the realness of the artificial players or correctly stating the goal of the study.

The final sample comprised *N* = 365 participants with an average age of 46 years (*SD* = 16.03). 47% of the participants categorized themselves as female. 19% stated having a bachelor’s or higher educational degree. Further sample characteristics are presented in Supplementary Material 1.

### 2.2 Design, manipulation, and game scenario

Participants played an online repeated 3-person prisoner’s dilemma game with the two co-players being pre-defined behavioral sequences. To make the conflict in the prisoner’s dilemma more concrete, it was translated into a real-world situation, called the “Concert Game.” Participants were asked to imagine that they are aiming to become professional pianists preparing for their first concert. To prepare for the concert, they have rented a practice room with a piano identical to the one at the concert. However, their practice room shares non-soundproof walls with two other piano rooms, which are used by two people who are also preparing for public performances. Practicing each day with the same two others, participants could choose each day between two behavioral actions: Practicing loudly, disturbing others (representing defection) or practicing with headphones (representing cooperation). When practicing with headphones, others are not disturbed, yet the sound is produced electronically. Hence, it prevents from practicing certain subtleties and limits the learning achievement. However, practicing is much more disturbed when others practice loudly in the neighboring rooms. This represents the typical characteristics of a prisoner’s dilemma game. The payoff matrix of the Concert Game is provided in Supplementary Table S1.

**Figure 2:**
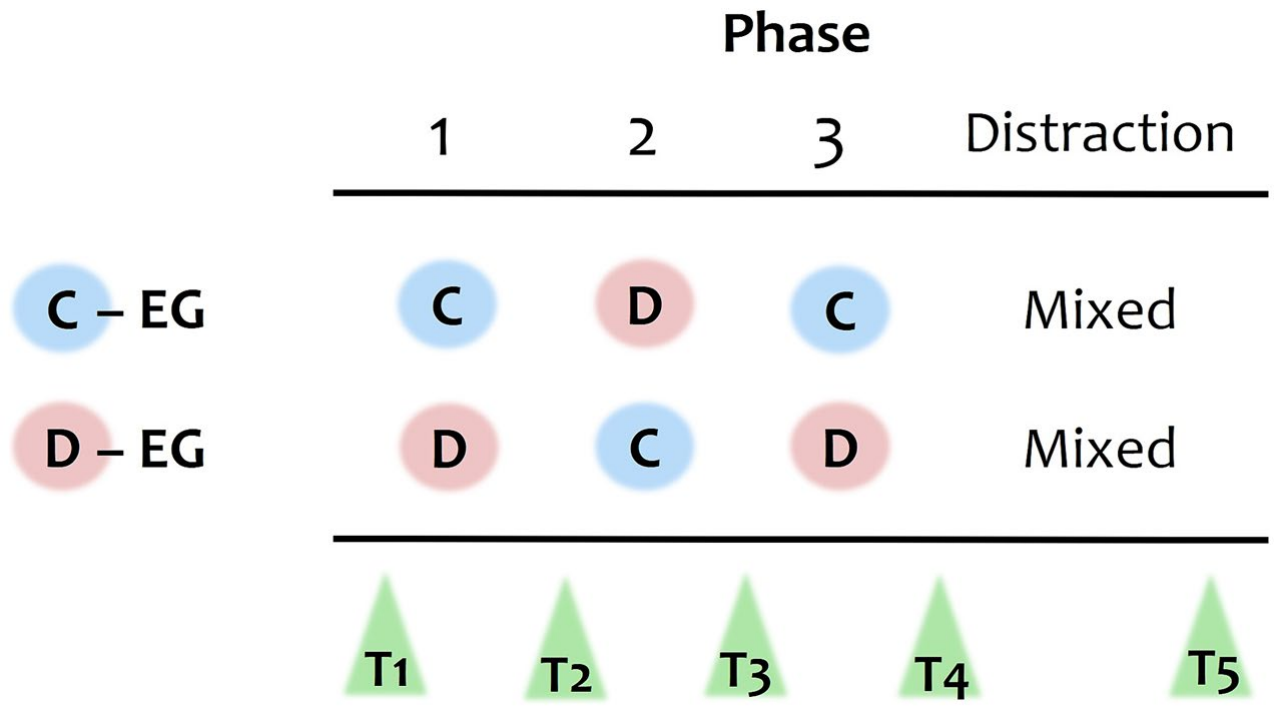
Operationalization of experimental groups. The prisoner’s dilemma game differed between the cooperative (C-EG) and defective experimental group (D-EG) in the order of their phases. Each experimental group consisted of three phases, characterized by either a cooperative setting (C), in which the artificial players cooperated, or a defective setting (D), in which they defected. The distraction phase was characterized by a mixed setting (Mixed), in which one of the artificial players cooperated and the other defected. Social and personal norms were assessed before the game (T1) and roughly after each phase at T2 – T5. The exact game setup of the 17 rounds is shown in Supplementary Figure S1.

The experimental design was a 2 (between-subjects factor *group*: cooperative vs. defective) x 5 (within-subjects factor *time*: T1 – T5) mixed design. The between-subjects factor experimental group varied the overall degree of cooperative actions of the artificial co-players. In the cooperative experimental group, the majority of the artificial players’ actions was cooperative, in the defective experimental group, the majority of the artificial players’ actions was defective (see Figure 2). Participants were randomly assigned to groups using simple randomization.

Within each experimental group, the cooperativeness of the social setting was varied repeatedly. Participants experienced different phases of the game, being each several rounds. In the cooperative group, the phases were cooperative-defective-cooperative (C-D-C): five rounds in a cooperative setting, three rounds in a defective setting, and four rounds in a cooperative setting. In the defective group, the phases were reversed to defective-cooperative-defective (D-C-D, see Figure 2). In between two phases, there was each one round of a mixed setting with one of the artificial players cooperating and the other defecting, to make transitions more realistic. After these three phases, a distraction phase followed in both groups, being three rounds of a mixed setting. In total, participants played the game for 17 rounds, being unaware of the final duration (for the exact setup of the 17 rounds of the game, see Supplementary Figure S1). The distraction phase was supposed to lead to moderate social norms in both groups, before testing for behavioral differences between groups after the game. At in total five measurement time points, participants were asked to state their norms: Before (T1) and (roughly) after each phase (T2 – T5).

### 2.3 Measures

All materials are shown in Supplementary Material 4. If not indicated otherwise, items were presented with a response slider ranging from 1 “not agree at all” to 101 “absolutely agree.” The norm scales as well as the manipulation check scale were created by taking the mean of two items, one for each behavioral option in the game (i.e., playing the piano via headphones vs. playing loudly). All items directed toward defectivity (i.e., playing loudly) were reversed beforehand. Hence, higher values indicate stronger cooperativeness. Internal consistencies for the scales at each measurement time point are presented in Supplementary Table S2.

#### 2.3.1 Trait cooperativeness (at T1)

The slider measure of social value orientation (Murphy et al., 2011) was applied for the trait cooperativeness measure. Participants were asked to allocate hypothetical money to themselves and to another unknown person. The money is allocated using a single slider for both allocations. The amounts of money received by the person herself and the other person are displayed above and underneath a slider, changing dynamically when the slider is moved. Participants were presented with an example and then asked to make six money allocations. The trait cooperativeness scale was created according to the instructions given by Murphy et al. (2011). Higher values indicate a stronger motivation to cooperate.

#### 2.3.2 Social norms (at T1 – T5)

(Other-oriented) social descriptive norms were assessed via two items, such as “The others mostly play the piano via headphones.” (Other-oriented) social injunctive norms were assessed via: “The others believe that they should play the piano via headphones.” and the inverted item for the non-cooperative option. For exploratory purposes, self-oriented social norms were additionally assessed. Self-oriented social descriptive norms were assessed via two items, for instance: “The others believe that I mostly play the piano via headphones.” Self-oriented social injunctive norms were assessed via: “The others believe that I should play the piano via headphones.” and its inversion. Items for social descriptive norms at T1 slightly differed, assessing expectations (see Supplementary Material 4).

#### 2.3.3 Personal norms (at T1 – T5)

Other-oriented personal norms were assessed via: “I am deeply convinced that the others should play the piano via headphones.” and its inversion. Self-oriented personal norms were assessed via: “I am deeply convinced that I should play the piano via headphones.” and its inversion.

#### 2.3.4 Manipulation check (at T2 – T5)

Two items were used to indicate a successful manipulation, such as: “In the past 2 days, the others have mostly played the piano via headphones.”

#### 2.3.5 Follow-up behavior (at T5)

After the game, a follow-up behavior was assessed, measuring an aggregated behavior across multiple decisions, via the item: “Please decide now on how you will practice the next 5 days.” Participants were asked to indicate their cooperative behavior (i.e., practicing via headphones) from 0 to 5 days.

#### 2.3.6 Perceived realness of the game scenario (at T5)

Three items assessed how well participants could imagine themselves being a pianist practicing for a concert. An example item is: “During the game, the scenario felt very real to me.”

#### 2.3.7 Supposed goal of the study and credibility of the cover story (at T5)

In an open question, participants were asked to express their thoughts on the study’s goals. Credibility of the cover story was assessed via an open question on perceiving anything as odd during the game.

#### 2.3.8 Demographics (at T5)

Age, gender, German language proficiency, education, occupation, income, and political orientation on the left–right spectrum were assessed.

### 2.4 Procedure

The study was conducted as an online study via the platform SoSci Survey (Leiner, 2019). The study was announced as an online game, called the “Concert Game,” as part of a study. To strengthen the cover story of a real-time online game, the link to participate in the study was only active between 8 am and 10 pm so that online matching of participants would seem likely. Completing the study took participants on average 21 min (*SD* = 6.60).

#### 2.4.1 Before the game

In the beginning, participants were welcomed and gave their informed consent. Next, trait cooperativeness was assessed. Then, the 3-person prisoner’s dilemma game, called the “Concert Game,” was explained. Participants received full information on the payoff matrix of the game and were presented with an example round. To ensure that participants read and understood the game instructions, five previously announced, multiple choice comprehension questions followed (see Supplementary Material 4). If more than one question was answered incorrectly, game instructions were presented again. If for a second time, more than one of the same comprehension questions was answered incorrectly, participation was terminated. That was the case for 92 participants. The other participants were then asked to rate their social descriptive, social injunctive and personal norms for a first time (T1). Before the game started, to convey the impression of an online game, participants were supposedly matched in a group with two other participants. Participants were presented with a progress bar and told that matching took on average 4 min. After 1 min, two more participants supposedly had joined the group and participants were able to start the game.

#### 2.4.2 The concert game

The game was explained as follows:

*Please imagine the following scenario: You are a passionate musician. Your instrument is the piano. In a while you have an important performance; you play your first big concert. This concert is decisive for your future career as a pianist. To prepare for the concert, you have rented a practice room with a piano identical to the one you will play at the concert for 3 h per day.*

*Unfortunately, your practice room is located in a triangle with two other practice rooms, which are used by two people who are also preparing for public performances. The walls of the rooms are very thin, so that you can hear each other practicing. If you play the piano loudly, you disturb the others while practicing. Likewise, if the others play loudly, you will be disturbed. Therefore, all pianos have the option of being played electronically via headphones. This way, others are not disturbed, but the sound production is not the same when playing electronically via headphones, which prevents you from practicing certain subtleties. So via headphones your learning achievement is somewhat limited, but nowhere near as much as when disturbed from the loud practicing of others in the neighboring practice rooms.*

*You will have to practice with the same two people in the time to come. Every day you may decide anew whether you want to practice with headphones or loudly.*

Participants played 17 rounds of the Concert Game. Each round consisted of a decision page, a holding page, and a feedback page. On the decision page, participants were presented with the choice of practicing with headphones versus practicing loudly. For all decisions, the information on the payoff matrix was presented. To simulate semblance with an online game, after each decision, participants were directed to a holding page, having to wait between 0 and 10 s for the artificial players before being able to continue (with longer waiting times in the beginning of the game and after questionnaires). The feedback page showed a table with all players’ practice behaviors of the current round, the practice points for the current round and the total collected points. Participants were given no information on the number of rounds played or upcoming. During the game, at T2 – T4 social norms, personal norms, and a manipulation check were assessed.

#### 2.4.3 After the game

After the last round of the game, which participants were not aware of, the same questionnaire as during the game was presented, assessing all types of norms and the manipulation check (T5). Afterwards, participants were told that the game would proceed, and a follow-up behavior was assessed, representing a more aggregated form of behavioral decision (see Section 2.3). Subsequently, the following variables were assessed in the presented order: perceived realness of the game scenario, supposed goal of the study, credibility of the cover story, and demographics. Finally, participants were informed that they had successfully practiced for the concert, debriefed, and dismissed.

### 2.5 Analysis plan

As preliminary analysis, the effectiveness of the experimental manipulation is investigated via testing whether participants experienced the different phases within the game as significantly differently depending on the group. A mixed analysis of variance is conducted with the within-subject factor time (T2 vs. T3 vs. T4 vs. T5), the between-subject factor group (C-EG vs. D-EG), and the manipulation check as dependent variable. A significant interaction effect will indicate a successful implementation of group differences. Further, via a set a planned contrasts it is tested whether each phase within the game was perceived as significantly differently to the one before and after, depending on the group. Three repeated measures contrasts are defined, comparing T2 to T3, T3 to T4, and T4 to T5. Significant interaction effects between contrast and group are analyzed.

Hypothesis 1.1 stated differences in the rates of change in social and personal norms. For that, difference variables for each norm between single consecutive measurement time points are calculated and aggregated across time. Difference variables of social and personal norms are then compared via a repeated measures orthogonal contrast using a multilevel model approach. Hypotheses 1.2 and 1.3 addressed the assumed seasonal change in social norms and linear change in personal norms. Both hypotheses are investigated via a multilevel model approach, defining for each mixed model two factors: (1) the between-subject factor group (C-EG vs. D-EG) and (2) a contrast for the within-subject factor time (T1 vs. T2 vs. T3 vs. T4 vs. T5), describing the assumed development (i.e., a seasonal change or a linear trend).

Hypotheses 2.1 and 2.2 assumed that social norms are solely explained by the experimental group, while personal norms are additionally predicted by trait cooperativeness and the interaction term. Both hypotheses are tested via multiple regressions, including social or personal norms as dependent variables and the experimental group, trait cooperativeness, and their interaction as predictors.

Similarly, Hypothesis 3, stating the additional influence of personal norms over and above the influence of social norms on the follow-up behavior, was addressed via multiple regression.

In Hypothesis 4.1, a group difference in personal norms at T5 was assumed, meaning that personal norms in the cooperative group were expected to be more cooperative than in the defective group. Similarly, Hypothesis 4.2 stated a group difference in the follow-up behavior. All group differences are investigated via one-tailed Welch’s *t*-tests.

## 3 Results

Multiple comparisons were accounted for by correcting alpha error rates with Bonferroni-adjustments by the number of tests conducted on the same null hypothesis (i.e., *α* = 0.05/[number of tests], Rubin, 2017). The analysis plan was preregistered^2^; any deviations are made explicit. Additional analyses are presented in a separate paragraph in each section and introduced as exploratory. Data were analyzed using R, version 4.1.3. The data that support the findings of this study are openly available in Open Science Framework at doi: 10.17605/OSF.IO/4CZ2B.

### 3.1 Manipulation check

To examine whether the experimental manipulation was successful, and participants experienced the different phases within the game as significantly differently depending on the group, a mixed analysis of variance on the manipulation check was conducted first. The interaction effect of the within-subject factor time (T2 vs. T3 vs. T4 vs. T5) and the between-subject factor group (C-EG vs. D-EG) was significant, *F*(3,1089) = 564.81, *p* < 0.001, *η_p_^2^* = 0.61. Second, through a set of planned contrasts, it was investigated whether consecutive phases within the game were perceived as significantly differently, depending on the group. All contrast analyses yielded significant interaction effects between contrast and group (*ps* < 0.001). Hence, participants perceived each phase as significantly differently to the one before and after, depending on their group.

### 3.2 Differences in rates of change

To test for different rates of change between social and personal norms, difference variables for each norm (i.e., self-oriented personal norms, other-oriented personal norms, social descriptive norms, and social injunctive norms) between single consecutive measurement time points were calculated and aggregated across time. Using a multilevel model approach, a repeated measures orthogonal contrast [1 1–1 −1] for the type of norm was defined, comparing both social norms to both personal norms regarding their differences between measurements. The orthogonal contrast revealed that social norms changed significantly more between measurements than personal norms [*B* = 10.70, *t*(1094) = 28.23, *p* < 0.001, *r* = 0.65], supporting Hypothesis 1.1.^3^ Hence, social norms showed a higher rate of change than personal norms.

**Figure 3:**
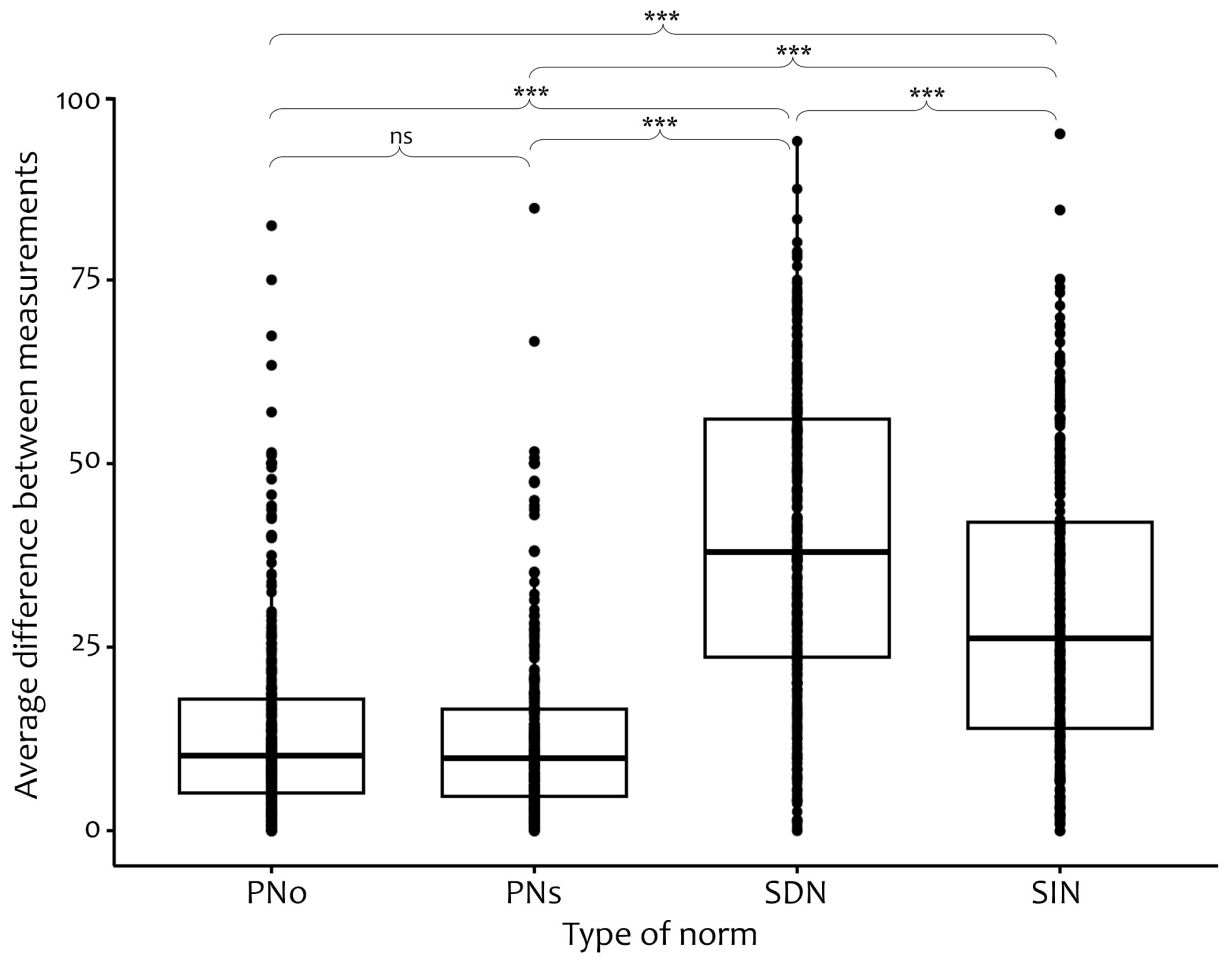
Average differences between measurements in personal and social norms. Difference variables are averaged across differences between consecutive measurements (i.e., T1–T2, T2–T3, T3–T4, and T4–T5). PNo, other-oriented personal norm; PNs, self-oriented personal norm; SDN, social descriptive norm; SIN, social injunctive norm. ^***^p < 0.001.

Exploratory, pairwise comparisons (*t-*tests with Bonferroni adjustment) between the difference variables of all four types of norms were conducted, illustrated in Figure 3. Apart from self-oriented and other-oriented personal norms (*p* = 1), all *post hoc* tests resulted to be significant (*ps* < 0.001) with social descriptive norms changing the fastest.

### 3.3 Seasonal change in social norms vs. linear change in personal norms

Next, seasonal change in social norms and linear change in personal norms was investigated, using a multilevel model approach. For each mixed model, the between-subject factor group (C-EG vs. D-EG) and a contrast for the within-subject factor time (T1 vs. T2 vs. T3 vs. T4 vs. T5) was defined.

First, it was tested whether social norms changed seasonally [−1 3 –4 3 –1] with the different phases of the game depending on the group, by defining separate models for each social descriptive and social injunctive norms. The interaction effect of seasonal contrast and group showed to be significant for both social descriptive norms [*B* = −6.63, *t*(1458) = −36.02, *p* < 0.001, *r* = −0.69] and social injunctive norms [*B* = −4.24, *t*(1458) = −22.87, *p* < 0.001, *r* = −0.51], confirming Hypothesis 1.2 that social norms change seasonally with the cooperativeness of the social setting (see Supplementary Table S3).

**Figure 4:**
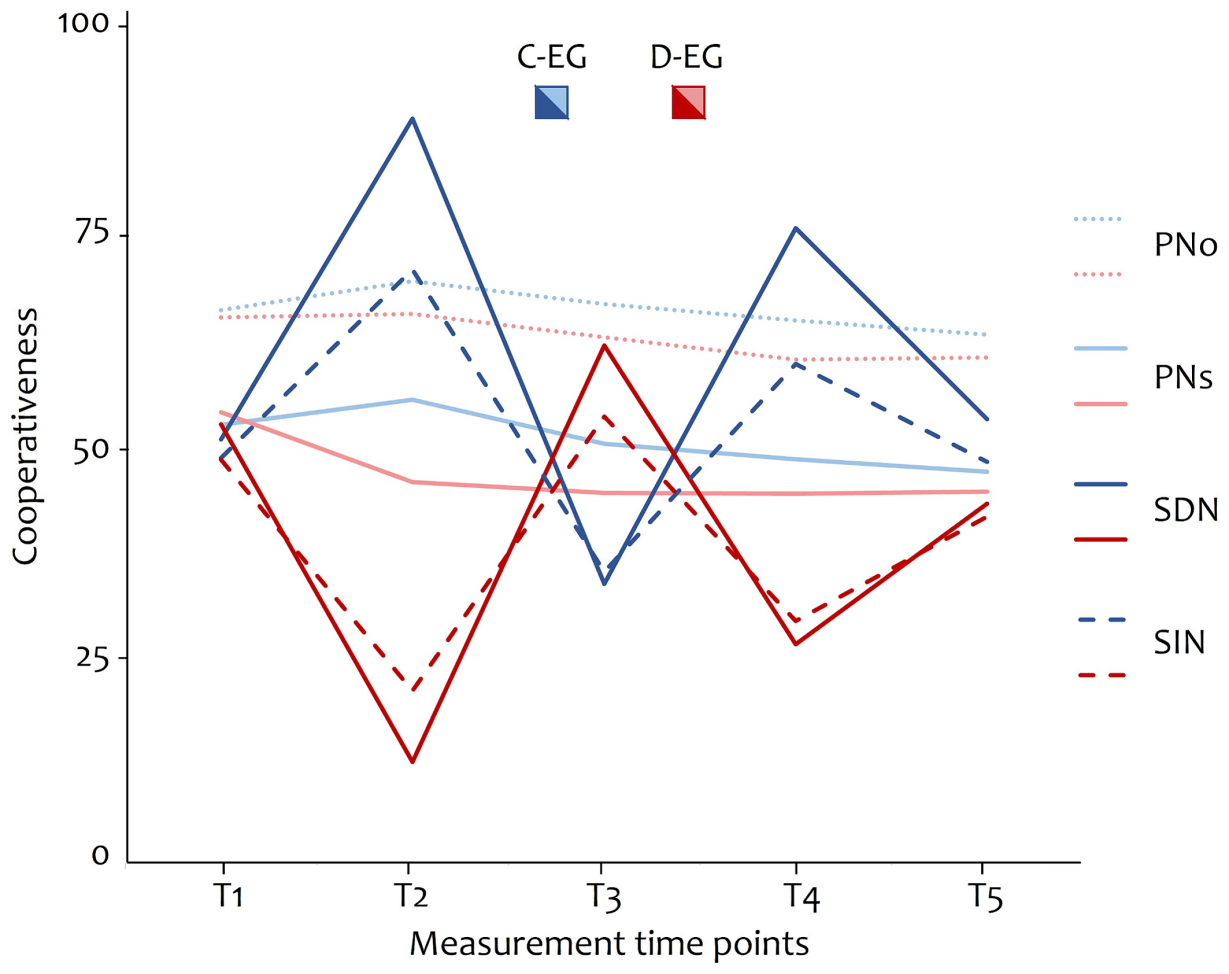
Changes in the cooperativeness of social and personal norms depending on the group. C-EG, cooperative group; D-EG, defective group; PNo, other-oriented personal norms; PNs, self-oriented personal norms; SDN, social descriptive norms; SIN, social injunctive norms.

For change in personal norms, a linear trend contrast was set for the factor time [−2 –1 0 1 2]. Again, two separate models for the dependent variables self-oriented and other-oriented personal norms were defined. The interaction effect of the linear trend contrast and the group showed to be non-significant for both self-oriented personal norms [*B* = 0.10, *t*(1458) = 0.37, *p* = 0.713, *r* = 0.01] and other-oriented personal norms [*B* = 0.22, *t*(1458) = 0.79, *p* = 0.428, *r* = 0.02]. However, there was a significant main effect of the linear trend in both self-oriented personal norms [*B* = −1.90, *t*(1458) = −7.17, *p* < 0.001, *r* = −0.18] and other-oriented personal norms [*B* = −1.26, *t*(1458) = −4.57, *p* < 0.001, *r* = −0.12]. Results are presented in Supplementary Table S4. Hence, the assumed linear trend in personal norms depending on the group was not confirmed (Hypothesis 1.3). Personal norms rather decreased linearly in both groups. Figure 4 shows the change over time in all four types of norms.

Exploratory, group differences in social norms at T5 were examined (two-sided *t*-tests). Although the preceding distraction phase, was supposed to “neutralize” social norms, both social norms still differed between groups at T5 (*ps* < 0.001).

### 3.4 Predictors of social and personal norms

Using multiple regression analysis, it was investigated whether social norms were solely explained by the experimental group (see Supplementary Table S5), whereas personal norms were additionally explained by trait cooperativeness and the interaction term (see Supplementary Table S6). As predicted in Hypothesis 2.1, social injunctive and social descriptive norms were solely predicted by the experimental group (*ps* < 0.001). Contrary to Hypothesis 2.2, self- and other-oriented personal norms were neither explained by the group, nor trait cooperativeness or the interaction.

Exploratory, regression analyses on self-oriented and other-oriented personal norms with trait cooperativeness, the experimental group, self-oriented, and other-oriented social norms as predictors were conducted (see Supplementary Table S7). By including social norms as predictors into the analysis, both trait cooperativeness and the group in addition to all social norms showed to significantly predict self-oriented personal norms. Self-oriented social descriptive norms were the strongest predictor of self-oriented personal norms. Other-oriented personal norms were solely predicted by self-oriented social injunctive norms.

### 3.5 Predictors of behavior

Testing the influence of personal norms on the follow-up behavior (Hypothesis 3), a regression analysis with social and personal norms as predictors was conducted (see Table 1).^4^ Solely self-oriented personal norms significantly predicted behavior after the game.

**Table 1:** Regression of follow-up behavior on social and personal norms.

	*R*^2^_adj_	*B*	β	*t*	*F*	*p*
Model	0.35				50.09	<0.001^***^
Social descriptive norm		0.01	0.07	1.35		0.178
Social injunctive norm		−0.00	−0.00	−0.06		0.950
Self-oriented personal norm		0.04	0.58	12.20		<0.001^***^
Other-oriented personal norm		0.00	0.02	0.53		0.593

*N* = 365. ^***^p < 0.001.

### 3.6 Group differences in personal norms

Addressing Hypothesis 4.1 that personal norms are more cooperative in the cooperative than defective group after the game, personal norms at T5 were compared between groups. Descriptively, self-oriented personal norms at T5 were slightly more cooperative in the cooperative (*M_C-EG_* = 46.05, *SD_C-EG_* = 26.25) than the defective group (*M_D-EG_* = 43.70, *SD_D-EG_* = 26.68), while the difference did not result to be of significance in a one-tailed Welch’s *t*-test [*t*(362) = 0.85, *p* = 0.199, *δ* = 0.09]. Similarly, the descriptive difference between groups regarding other-oriented personal norms (*M_C-EG_* = 62.16, *SD_C-EG_* = 24.23; *M_D-EG_* = 59.47, *SD_D-EG_* = 26.82) was not significant [*t*(363) = 1.01, *p* = 0.157, *δ* = 0.11].

Exploratory, group differences in personal norms throughout the game were investigated, using one-tailed Welch’s *t*-tests (see Supplementary Table S8). Self-oriented personal norms were significantly more cooperative in the cooperative than defective group at T2 (*p* < 0.001, *δ* = 0.33). Other-oriented personal norms did not differ between groups at any point in time. Additionally, differences in personal norms within each group before and after the game (i.e., between T1 and T5) were explored in paired, two-sided Welch’s *t*-tests. After correcting for multiple comparisons, self-oriented personal norms decreased throughout the game in both groups (*ps* < 0.01), while other-oriented personal norms missed the corrected alpha level (*p_C-EG_* = 0.065, *p_D-EG_* = 0.021).

### 3.7 Group differences in behavioral decisions

**Figure 5:**
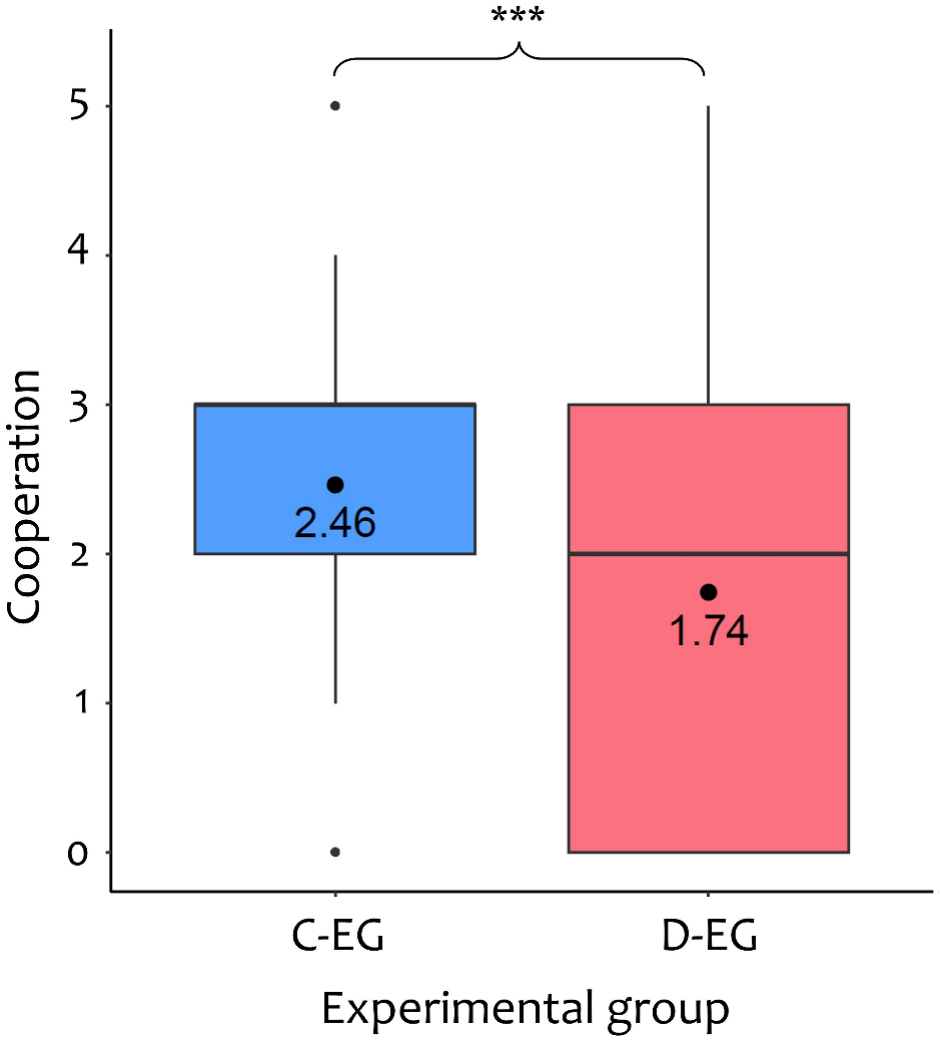
Cooperation in the follow-up behavior depending on the experimental group. Group medians are indicated by the black lines, means by the black points and their values given underneath. Significance is calculated by a one-tailed Welch’s *t*-test. C-EG, cooperative group; D-EG, defective group. ^***^p < 0.001.

As predicted in Hypothesis 4.2, participants in the cooperative group cooperated significantly more in the follow-up behavior after the game than those in the defective group, shown in a significant one-tailed Welch’s *t*-test [*t*(362) = 4.53, *p* < 0.001, *δ* = 0.47], as indicated in Figure 5.

### 3.8 Self-oriented vs. other-oriented social norms

**Figure 6:**
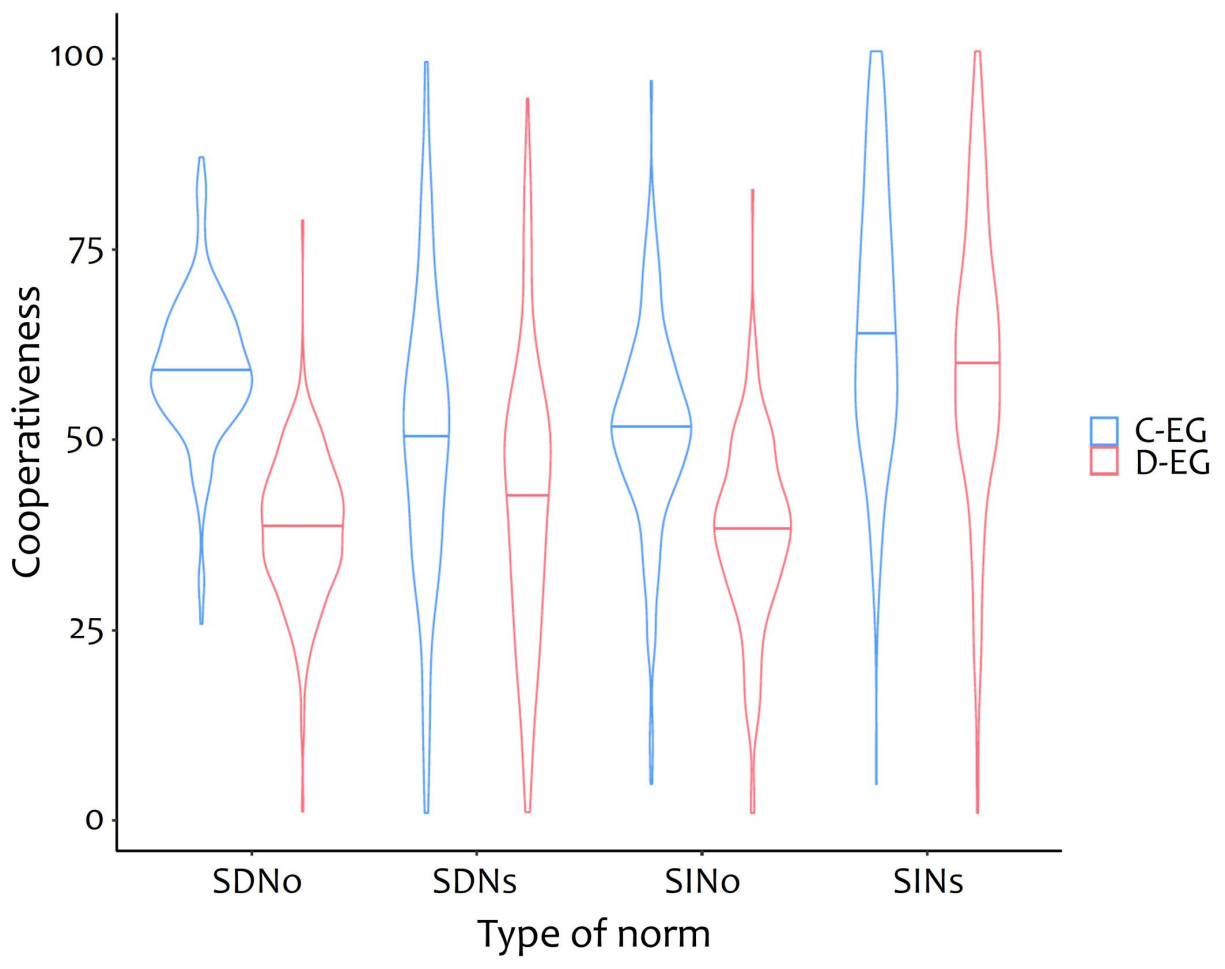
Cooperativeness of different types of social norms split by experimental group. C-EG, cooperative group; D-EG, defective group. SDNo, other-oriented social descriptive norm; SDNs, self-oriented social descriptive norm; SINo, other-oriented social injunctive norm; SINs, self-oriented social injunctive norm. Horizontal lines show medians.

Exploratory, differences between self-oriented social norms and other-oriented social norms were analyzed. Figure 6 shows the cooperativeness of different types of social norms, indicating that self-oriented social norms, meaning what others expect I do or approve of, have a greater variability. Using Levene tests, differences in variances were examined in six pairwise comparisons. Variances of all self-oriented social norms differed significantly from those of other-oriented social norms (*ps* < 0.001), whereas the variances of the two social norms of the same orientation did not differ. To further investigate the assumption that self-oriented social norms are stronger subject to subjectivity, the different social norms were correlated with trait cooperativeness, resulting to be significant only for self-oriented social descriptive norms (*r* = 0.25, *p* < 0.001).

## 4 Discussion

The present work aimed at contributing to a better understanding of change in individuals’ norms. Norms have been of great interest to social psychologists and many others; however, dynamic norm processes are yet little understood (van Kleef et al., 2019; Andrighetto and Vriens, 2022). In the present work, an experimental setting that allowed investigating differences in the temporal dynamics of social and personal norm change was introduced and assumptions on differences in norm change were addressed. Social norms were assumed to be adapted quickly whenever the social situation changes, while personal norms were expected to change more slowly and gradually, depending not only on situational but also on personal factors (*cf.* Batzke and Ernst, 2023b). To investigate the assumed differences, participants played a repeated prisoner’s dilemma game with artificial co-players. Therein, the cooperativeness of the social setting (i.e., the co-players behavior) changed repeatedly *within* each experimental group, which was assumed to result in participants quickly adapting their social norms. Moreover, the overall cooperativity of the artificial co-players differed *between* groups, which was assumed to show in slow adaptations in participants’ personal norms. Group differences in personal norms were assumed to affect behavioral decision-making. In the following, results relating to the assumed underlying qualitatively different processes of different norms (Section 4.1), influences on personal norms (Section 4.2), the influence of personal norms on cooperation (Section 4.3) as well as limitations and future work concerning the questions of whether and how personal norms change (Section 4.4) are discussed. Section 5 concludes.

### 4.1 Qualitatively different processes in social and personal norms and their influence on decision-making

Based on the experimental results, one may assume different temporal dynamics in social and personal norms, potentially indicating qualitatively different processes of norm change. As predicted, social descriptive and social injunctive norms had higher rates of change than personal norms (supporting Hypothesis 1.1). They were adapted repeatedly according to the cooperativeness of the social setting showing in seasonal changes (supporting Hypothesis 1.2). Moreover, social norms were solely explained by the experimental group (supporting Hypothesis 2.1). Hence, hypotheses regarding social norms were largely supported, suggesting that changes in the social environment were observed and accounted for immediately (Schultz et al., 2007; Nolan et al., 2008). Social descriptive and social injunctive norms may change repeatedly within a short timeframe, being strongly context dependent. It can therefore be concluded that social norm adaptation is fast and quickly reversible. Unlike social norms, personal norms were assumed to change linearly, developing toward cooperativeness or defectivity (depending on the group), which could not be supported (contradicting Hypothesis 1.3). The results rather indicated that personal norms trended toward defectivity in both groups. A group difference in personal norms was only found at T2 with a small-medium sized effect, but significance disappeared after T2 (contradicting Hypothesis 4.1). Different interpretations for these results are discussed in Section 4.4.

### 4.2 Influences on personal norms

Psychological research so far has identified several associated factors of personal norms such as social norms, ascription of responsibility, problem awareness, guilt, and so forth. (see Stern et al., 1999; Bamberg and Schmidt, 2003; De Groot and Steg, 2009). In the present work, it was assumed that situational and personal variables as well as their interaction affect personal norm change. Neither the experimental group, nor trait cooperativeness, nor their interaction resulted to be of significance in the preregistered analysis (contradicting Hypothesis 2.2). However, exploratory analyses revealed that the influence of both predictors might have been masked. By adding social norms to the analysis, the group, the personality factor trait cooperativeness as well as different types of social norms explained variance in self-oriented personal norm. This supports the assumption of situational and personal factors influencing personal norm change and the idea of an underlying more complex process (compared to social norms change).

While existing research showed that (other-oriented) social norms and personal norms are associated (e.g., Bamberg et al., 2007; Bamberg, 2013), the present results suggest that variance in personal norms is particularly well predicted by *self-oriented* social norms. Hence, what an individual perceives as expectations of others regarding the *own* behavior (vs. general behaviors of others) is strongly related to the individual’s personal norms. Moreover, personal norms (as well as self-oriented social norms) showed to be related to trait cooperativeness. Based on these results, personal norms cannot be assumed to merely result from a general cooperative personality and to lack “social conditionality,” as stated by Bicchieri and Dimant (2019), defining a “moral rule” as *not* depending “on others doing X [i.e., a behavior] or thinking that you should do X.” (p. 447f, text in square brackets added). Rather the idea found support that personal norms are learned expectations of others in between purely external social factors (such as other-oriented social descriptive norms) and internal personality factors. This relates to the concept of internalization of social norms into personal norms (Thøgersen, 2006).

Accordingly, self-oriented social norms might be in-between personal and other-oriented social norms as they were less univocally given by the situation than other-oriented social norms (with their variances being significantly larger). Thus, self-oriented social norms can be assumed to be more determined by subjectivity as they partly correlated with trait cooperativeness. Since they were strongly related to personal norms, others’ expectations regarding the individual (and not people in general) could be a promising leverage point for future norm-based intervention studies. So far, norm-based intervention research has focused on *other-oriented* social norms under the terms of *social norm information and feedback* (Abrahamse and Steg, 2013), *social norms marketing* (Miller and Prentice, 2016), or *social norm nudges* (Sunstein, 2014).

### 4.3 Influence of personal norms on cooperation

Looking at the influence of personal norms on cooperation, the follow-up behavior was predicted only by self-oriented personal norms (but not social norms, only partly supporting Hypothesis 3). Moreover, participants in the cooperative group cooperated significantly more (supporting Hypothesis 4.2). In line with existing research, personal norms showed to be highly relevant for behavioral decisions (*cf.* Hunecke et al., 2001; Onwezen et al., 2013; Han, 2014). Beyond that, relating work supports the finding that personal norms have an advantage in explaining cooperative behavior in social dilemmas over social norms (Catola et al., 2021). Capraro and Rand (2018) created an experimental situation in which the personal norm and social descriptive norm conflicted and found that people tended to follow their personal norm. One might reason that the stronger influence of personal norms (vs. social norms) on cooperation is due to a justification/rationalization process (Steele, 1988; Sherman and Cohen, 2002). Accordingly, participants might have preferred to justify their decisions via personal norms as people generally want to perceive themselves as self-consistent. Hence, participants might have adapted their personal norms prior to their decisions, which would explain the strong influence that personal norms had on cooperation. The great explanatory power that personal norms have on behavior was repeatedly shown in research (for reviews see Capraro and Perc, 2021; Capraro et al., 2024). It yet remains for future research to further investigate the mechanisms underlying personal norm change and to explore the dynamic interplay of personal and social norms. As argued by Tverskoi et al. (2023), “one can hardly understand social behavior without understanding the dynamics of personal [normative] beliefs” (p. 10), text in square brackets added.

Throughout all analyses other-oriented personal norms (i.e., an individual’s beliefs about what *others* should do) were less affected by other variables and had close to no effects on behavioral decision-making. Contrary, self-oriented personal norms (i.e., an individual’s beliefs about what *itself* should do) were strongly predictive of behavior and at least temporarily affected by the experimental manipulation. The missing specification on self-oriented personal norms is one possible explanation, why previous research on norm change assessing personal norms as a self−/other-combination found no change in personal norms (Szekely et al., 2021; Bicchieri et al., 2022).

### 4.4 Do personal norms change, and how? – limitations and future work

While social norms did change with the cooperativeness of the social setting, the more interesting questions remained inconclusive: Do personal norms change, and how? There are two possible answers to that question. On the one hand, one may assume that personal norms remain rather stable throughout the lifetime with major changes largely happening in childhood and early adulthood (*cf.* Turiel, 1983; Nucci, 2001). Based on this assumption, the shown group difference in personal norms could be attributed to differences in the activation level, in line with Schwartz (1977) *norm activation model* (see also Schwartz and Howard, 1981, 1982) and its extension the *belief-value-norm theory* (Stern et al., 1999; Stern, 2000). Accordingly, self-oriented personal norms might have been activated by the respective social norm in the first phase of the game. The differences showed at T2 with personal norms being activated toward cooperation in the cooperative group that just experienced a cooperative setting and vice versa for the defective group. Furthermore, one could assume that people got frustrated after T2, which deactivated their personal norms, showing in a tendency toward central neutrality, indicating indifference. Being one of the most influential psychological theories on personal norms, the norm activation model has incited numerous studies on influencing factors of personal norm activation (Hunecke et al., 2001; Klöckner and Matthies, 2004; Bamberg et al., 2007; Han, 2014). Yet, to our knowledge few have addressed personal norm change as the theory is purely static, describing situational activation, not accounting for the possibility of norm change. But does that mean that personal norms are in fact static entities similar to traits?

Research has shown that people are highly adaptive, learn throughout their life, change their attitudes, values, and even personality traits (Bardi et al., 2009; Otto and Kaiser, 2014; Bleidorn et al., 2021). This leads us to the second possible answer: Personal norms do change – presumably over longer periods of time like change in attitudes or values. This supports the presented assumption of a slower adaptation process and calls for long-term studies of personal norms change. However, assuming slow change does still allow for observing an excerpt of personal norm change in shorter periods of time. Although it suggests that assessing change empirically may be challenging, as one is looking for small effects.

There are numerous potential reasons why the present study was unsuccessful in showing lasting change in personal norms. Possibly, the manipulation was too weak. As differences did show descriptively between groups, one could assume that stronger manipulations would increase the effect. In future work, a more existential game scenario than practicing the piano for a concert could be applied. Also, the personal norms measure might have been insensitive to the induced change. In the present study, personal and social norms were measured directly after decisions were made. As people want to perceive themselves as self-consistent (see Section 4.3), participants might have guessed their personal norms from their behavior in an attribution-like process about themselves (Bem, 1967, 1972). If participants did not change their behavior, they may have therefore indicated no change in their personal norms as well. Generally, indicating the own personal norms via self-report requires some level of introspection, and, doing so repeatedly within a short amount of time (game duration was about 20 min on average), requires a great amount of compliance. More indirect measures might improve validity, for instance assessing personal norms via the willingness to (costly) punish others who have violated a norm in line with Axelrod’s (1986) notion of norm internalization as increased incentive to punish norm violators.

While all the above may be (partly) accurate, why did the cooperative group not show a development toward more cooperativeness in personal norms? Possibly, participants got bored during the rather simple game and therefore did not change their personal norms toward the arguably more effortful direction of more cooperativeness. Potentially, prisoner’s dilemma games make learning cooperative personal norms difficult, as contextual variables are limited to a minimum. In the complexity of the real world, early stages of learning cooperative personal norms may be accompanied by attributing behavioral decisions to situational cues before a personal norm is generalized across single decisions. Situational cues are however limited in the simplified prisoner’s dilemma situation. This could also explain the descriptive decrease in the cooperativeness of personal norms in the social dilemma context found in previous work (*cf.* Szekely et al., 2021; Tverskoi et al., 2023).

Still, none of these explanations may account for the change in personal norms that participants showed in the cooperative group, increasing in cooperativeness to T2 and decreasing thereafter. An interesting and quite plausible explanation relates to *prospect theory* (Tversky and Kahneman, 1992), stating that negative experiences have a stronger impact than positive experiences. Therein, the authors described an asymmetry between losses and gains, stating that “losses loom larger than gains” (p. 298). Accordingly, the present results showed that personal norms in the cooperative group developed toward defectivity only *after* T2 (i.e., after the first encounter with defection), showing an asymmetrical development in self-oriented personal norms compared to the defective group. Experiencing defective co-players (i.e., making a negative experience) might have eroded participants’ personal norms – qualitatively differently to the positive impact of the prior cooperative setting (i.e., a positive experience). To test that assumption, an experimental group in which participants experience a longer cooperative or even purely cooperative setting would be necessary. So far, it remains unclear whether personal norm change is due to belief change or change in the activation level and how it can be directed toward more cooperativeness in the long run.

## 5 Conclusion

The present paper demonstrated an experimental approach to studying differences in the temporal dynamics of norm change processes. Assumptions were tested concerning the temporal dynamics of social and personal norm change. Results led to assume that social norms change faster and personal norms change slower. While the fast change in social norms was well predicted by situational changes, slow change in personal norms was multidetermined.

The present work aimed at taking a step toward better understanding norm change. Being able to truly grasp the potentials and limitations of norms in behavioral change, calls for knowledge about how, when, and why norms change. Yet, many questions particularly regarding personal norm change remain still open, among them: When do personal norms change, of what kind are the underlying mechanisms, how could results be used to foster learning cooperative personal norms, among others. These questions may inspire further research. Addressing them seems particularly relevant, as the significance of personal norms for behavioral decisions was demonstrated once again.

## Data availability statement

The datasets presented in this study can be found in the OSF repository at the following link: https://doi.org/10.17605/OSF.IO/4CZ2B. Further inquiries can be directed to the corresponding author.

## Ethics statement

Ethical approval was not required for the studies involving humans because the pilot study was already ethically approved. The studies were conducted in accordance with the local legislation and institutional requirements. The participants provided their written informed consent to participate in this study.

## Author contributions

MB: Conceptualization, Formal analysis, Methodology, Writing – original draft, Data curation, Visualization, Writing – review & editing. AE: Conceptualization, Methodology, Writing – review & editing.

## Footnotes

^1^ https://glimmpse.samplesizeshop.org/

^2^ https://osf.io/xgucf

^3^Upon further consideration, the preregistered first step in the analysis, meaning the analysis of variance testing for a main effect of type of norm, functioning as an omnibus test, was dropped due to no additional explanatory value.

^4^Although preregistered, the interaction terms were not included in the analyses to prevent overfitting.
