# Supplementary material for: Distinguishing fast change in social norms and slow change in personal norms in cooperative decision-making
Source: Front Psychol. 2024 May 30;15:1380341. doi: 10.3389/fpsyg.2024.1380341 (PMC11178139; doi:10.3389/fpsyg.2024.1380341)
Supplement: Supplementary file 1 [file Data_Sheet_1.pdf]

Supplementary Materials

1 Sample Characteristics

- *N* = 365
- 47% female, 52% male, 1% diverse
- Age: *M* = 46 (*SD* = 16): 21% 18 – 29 years, 17% 30 – 39 years, 18% 40 – 49 years, 20% 50 – 59 years, 20% 60 – 69 years, 5% 70 – 74 years
- Education: 27% no A-levels, 39% apprenticeship, 34% A-levels or higher
- Occupation: 12% students, 3% in apprenticeship, 52% employed, 4% self-employed, 5% unemployed, 20% retired, 4% incapacitated for work
- Income: 19% less than 1300€, 38% 1300 – 3200€, 25% 3200€ – 5000€, 9% more than 5000€ [8% missing values]
- Proficiency in German language: 4% fluent, 96% first language

2 Payoff Matrix of “The Concert Game”

Number cooperating	Defect payoff (i.e., practice points for practicing loudly)	Cooperate payoff (i.e., practice points for practicing with headphones)	Collective payoff
3	-	2	6
2	3	1	5
1	2	0	4
0	1	-	3

Supplementary Table S1. Individual and collective payoffs in the 3-person prisoner’s dilemma game (called “The Concert Game”) depending on the number of cooperating players.

3 Operationalization of Experimental Groups

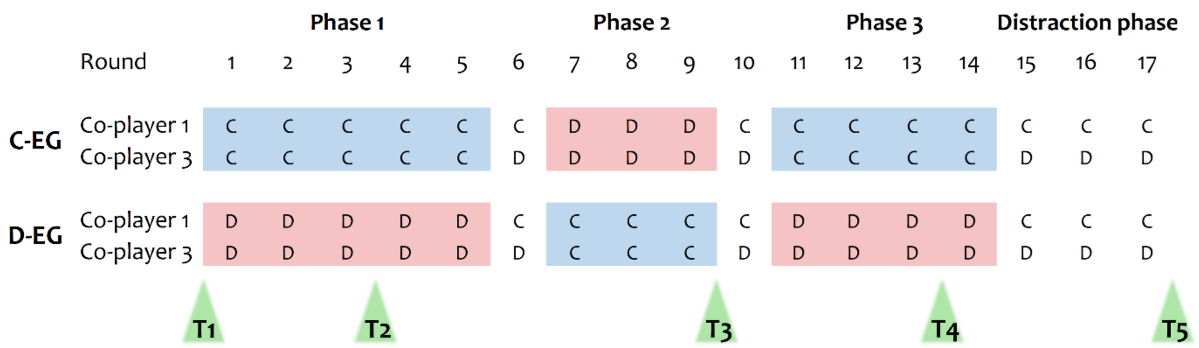

Supplementary Figure S1. Operationalization of experimental groups. Participants played the social dilemma game with two artificial co-players (1 and 3), themselves being player 2. The game differed between the cooperative (C-EG) and defective experimental group (D-EG) in the order of their phases. Each experimental group consisted of three phases, characterized by either a cooperation (blue color) or defection (red color) of the co-players, and a distraction phase, characterized by a mixed setting in which one co-player cooperated and the other defected (white color). In between phases, single rounds of a mixed setting were added to make the game more realistically. Social and personal norms were assessed before the game (T1) and roughly after each phase (after rounds 3, 9, 13 and 17) at T2 – T5.

4 Materials

Materials were shown to participants in German language and are below presented translated in English (originals can be found here: https://osf.io/xgucf). If not indicated otherwise, items were presented with a response slider ranging from 1 “not agree at all” to 101 “absolutely agree”. In addition to the below presented materials, the following measures were assessed at T5, but are not presented here, as they were not included in any analysis or hypothesis: Trustworthiness and predictability of the co-players, personal values, reactance, and strategies during the game. All materials are shown under: https://osf.io/xgucf.

Trait cooperativeness (at T1)

The slider measure of social value orientation (Murphy et al., 2011) was applied for the trait cooperativeness measure. Participants were asked to allocate hypothetical money to themselves (“You receive”) and to another unknown person (“The other person receives”). The money is allocated using a single slider for both allocations. The amounts of money received by the person herself and the other person are displayed above and underneath a slider, changing dynamically when the slider is moved. Participants were presented with an example and then asked to make six money allocations. The trait cooperativeness scale was created according to the instructions given by Murphy and colleagues (2011). Higher values indicate a stronger motivation to cooperate.

1.	You receive:	85	85
	The other person receives:	85	15
2.	You receive:	85	100
	The other person receives:	15	50
3.	You receive:	50	85
	The other person receives:	100	85
4.	You receive:	50	85
	The other person receives:	100	15
5.	You receive:	100	50
	The other person receives:	50	100
6.	You receive:	100	85
	The other person receives:	50	85

Comprehension of the game instructions (at T1)

1. The game is about...
    - preparing for a piano concert. (true)
    - preparing for a guitar concert. (false)

2. When I practice out loudly...
    - others are not disturbed. (false)
    - I have a harder time concentrating. (false)
    - I have a better practice experience. (true)
3. What of the following is true?
    - You play the game with four other people, with all practice rooms sharing thin walls. (false)
    - The concert is crucial for your future as a pianist. (true)
    - If it's just you playing the piano via headphones, you'll have the best practicing experience. (false)
4. What of the following is true?
    - Who plays in the practice rooms next door stays the same from day to day. (true)
    - When everyone is playing with headphones, no one can concentrate. (false)
    - If others are practicing loudly, it doesn't bother you. (false)
5. How many practice points you get...
    - is not dependent on the decisions of the other players. (false)
    - is determined each day based on the established awarding rules. (true)

Social norms (at T1 – T5)

Other-oriented social descriptive norms (at T1)

- The others will mostly play the piano loudly.
- The others will mostly play the piano via headphones.

Other-oriented social descriptive norms (at T2 - T5)

- The others mostly play the piano loudly.
- The others mostly play the piano via headphones.

Other-oriented social injunctive norms

- The others believe that they should play the piano loudly.
- The others believe that they should play the piano via headphones.

Self-oriented social descriptive norms (at T1)

- The others believe that I will mostly play the piano loudly.
- The others believe that I will mostly play the piano via headphones.

Self-oriented social descriptive norms (at T2 - T5)

- The others believe that I mostly play the piano loudly.
- The others believe that I mostly play the piano via headphones.

Self-oriented social injunctive norms

- The others believe that I should play the piano loudly.
- The others believe that I should play the piano via headphones.

Personal norms (at T1 – T5)

Other-oriented personal norms

- I am deeply convinced that the others should play the piano loudly.
- I am deeply convinced that the others should play the piano via headphones.

Self-oriented personal norms

- I am deeply convinced that I should play the piano loudly.
- I am deeply convinced that I should play the piano via headphones.

Manipulation check (at T2 – T5)

- In the past two days, the others have mostly played the piano loudly.
- In the past two days, the others have mostly played the piano via headphones.

Follow-up behavior (at T5)

- Please decide now on how you will practice the next five days.
  - Choice from 0 – 5 days

Perceived realness of the game scenario (at T5)

- I was able to empathize with the situation very well.
- I felt like actually being in the practice room scenario.
- During the game, the scenario felt very real to me.

Supposed goal of the study (at T5)

- What do you think was investigated in this study? [open question]

Credibility of the cover story (at T5)

- Did anything seem strange to you during the course of the study? [open question]

5 Internal Consistencies of Norm Scales at Different Measurement Time Points

	Measurement time point				
**Scale**	**T1**	**T2**	**T3**	**T4**	**T5**
Other-oriented social descriptive norm	0.61	0.94	0.83	0.90	0.56
Self-oriented social descriptive norm	0.48	0.85	0.81	0.80	0.81
Other-oriented social injunctive norm	0.63	0.87	0.75	0.79	0.54
Self-oriented social injunctive norm	0.58	0.82	0.71	0.80	0.73
Other-oriented personal norm	0.68	0.80	0.78	0.81	0.79
Self-oriented personal norm	0.75	0.81	0.80	0.80	0.79
Manipulation check	-	0.95	0.89	0.91	0.51

Supplementary Table S2. Internal consistencies of norm scales at different measurement time points. *N* = 365. T1 – T5 = measurement time points. Internal consistencies are calculated by Cronbach's alpha. The manipulation check was not assessed at T1.

6 Seasonal Change in Social Norms and Linear Change in Personal Norms

**Multilevel model of social descriptive norms**					
	df	*B*	*t*	*p*	*r*
Experimental group	363	10.46	21.19	< .001***	.74
Seasonal contrast	1458	-0.40	-2.17	.031*	-.06
Interaction	1458	-6.63	-36.02	< .001***	-.69
**Multilevel model of social injunctive norms**					
	df	*B*	*t*	*p*	*r*
Experimental group	363	6.82	10.30	< .001***	.48
Seasonal contrast	1458	-0.03	-0.14	.890	-.00
Interaction	1458	-4.24	-22.87	< .001***	-.51

Supplementary Table S3. Mixed multilevel model of social descriptive and social injunctive norms on the experimental group, a seasonal contrast for the factor time, and their interaction. $N = 365$. The seasonal contrast across the five measurement time points was defined as [-1 3 -4 3 -1]. *$p < .05$. ***$p < .001$.

**Multilevel model of self-oriented personal norms**					
	df	*B*	*t*	*p*	*r*
Experimental group	363	2.04	1.68	.094	.09
Linear contrast	1458	-1.90	-7.17	< .001***	-.18
Interaction	1458	0.10	0.37	.713	.01
**Multilevel model of other-oriented personal norms**					
	df	*B*	*t*	*p*	*r*
Experimental group	363	1.59	1.45	.147	.08
Linear contrast	1458	-1.26	-4.57	< .001***	-.12
Interaction	1458	0.22	0.79	.428	.02

Supplementary Table S4. Mixed multilevel model of self-oriented and other-oriented personal norms on the experimental group, a linear contrast for the factor time, and their interaction. $N = 365$. The linear contrast across the five measurement time points was defined as [-2 -1 0 1 2].
***p < .001.

7 Regressions of Social and Personal Norms

**Regression of social descriptive norms**						
	R^2_{adj}	*B*	β	*t*	*F*	*p*
Model	.55				151.20	***
Experimental group		-17.59	-.62	-8.68		***
Trait cooperativeness		0.12	.21	1.85		.066
Interaction		-0.08	-.24	-1.88		.060
**Regression of social injunctive norms**						
	R^2_{adj}	*B*	β	*t*	*F*	*p*
Model	.22				35.71	***
Experimental group		-10.72	-.37	-3.93		***
Trait cooperativeness		0.10	.17	1.15		.250
Interaction		-0.07	-.21	-1.23		.221

Supplementary Table S5. Regressions of social descriptive and social injunctive norms on experimental group, trait cooperativeness, and their interaction. $N = 365$. Alpha error corrected by the number of tests, e.g., $\alpha = .05 / 2 = .025$. ***$p < .0005$.

Regression of self-oriented personal norms

	R^2_{adj}	*B*	β	*t*	*F*	*p*
Model	.09				12.72	***
Experimental group		-5.97	-.13	-1.24		.214
Trait cooperativeness		0.21	.22	1.36		.176
Interaction		0.05	.09	0.50		.618

Regression of other-oriented personal norms

	R^2_{adj}	*B*	β	*t*	*F*	*p*
Model	.00				1.18	.319
Experimental group		-7.45	-.18	-1.66		.098
Trait cooperativeness		-0.17	-.20	-1.19		.237
Interaction		0.10	.21	1.08		.279

Supplementary Table S6. Regressions of self-oriented and other-oriented personal norms on experimental group, trait cooperativeness, and their interaction. $N = 365$. Alpha error corrected by the number of tests, e.g., $\alpha = .05 / 2 = .025$. ***$p < .0005$.

Regression of self-oriented personal norms

	R^2_{adj}	*B*	β	*t*	*F*	*p*
Model	.56				92.59	***
Experimental group		5.77	.12	3.10		.002**
Trait cooperativeness		0.13	.17	3.77		***
Other-oriented social injunctive norm		0.18	.11	2.65		.008*
Self-oriented social descriptive norm		0.78	.63	15.87		***
Self-oriented social injunctive norm		0.20	.16	4.38		***

Regression of other-oriented personal norms

	R^2_{adj}	*B*	β	*t*	*F*	*p*
Model	.37				42.99	***
Experimental group		2.53	.06	1.27		.206
Trait cooperativeness		-0.07	-.07	-1.52		.131
Other-oriented social injunctive norm		0.10	.07	1.30		.194
Self-oriented social descriptive norm		0.09	.08	1.70		.091
Self-oriented social injunctive norm		0.66	.58	13.53		***

Supplementary Table S7. Regressions of self-oriented and other-oriented personal norms on social norms, experimental group, trait cooperativeness as well as trustworthiness and predictability of the other players. $N = 365$. Due to multicollinearity (i.e., correlations between predictors above $r = .70$), the factor other-oriented social descriptive norm was excluded from both regressions. Alpha error corrected by the number of tests, e.g., $\alpha = .05 / 2 = .025$. *$p < .025$. **$p < .005$. ***$p < .0005$.

8 Group Differences in Self-Oriented and Other-Oriented Personal Norms at Different Measurement Time Points

**Self-oriented personal norms**					
	M (SD)		*t*	*p*	δ
	C-EG	D-EG			
T1	51.56 (24.68)	53.02 (25.75)	-0.55	.709	0.06
T2	54.49 (31.12)	44.81 (26.78)	3.18	< .001**	0.33
T3	49.35 (27.93)	43.54 (27.46)	2.00	.023	0.21
T4	47.51 (27.87)	43.48 (27.87)	1.38	.084	0.14
T5	46.05 (26.25)	43.70 (26.68)	0.85	.199	0.09
**Other-oriented personal norms**					
	M (SD)		*t*	*p*	δ
	C-EG	D-EG			
T1	65.04 (22.07)	64.17 (23.17)	0.37	.357	0.04
T2	68.46 (25.76)	64.61 (27.76)	1.37	.085	0.14
T3	65.74 (25.03)	61.86 (26.09)	1.45	.074	0.15
T4	63.82 (25.89)	59.24 (28.28)	1.61	.054	0.17
T5	62.16 (24.23)	59.47 (26.82)	1.01	.157	0.11

Supplementary Table S8. Group differences in self-oriented and other-oriented personal norms at different measurement time points**.** *N* = 365. One-sided Welch's t-tests. Degrees of freedom were

adapted according to Welch correction. C-EG = cooperative group, D-EG = defective group. δ = effect size Cohen's delta. Alpha error corrected by the number of tests, e.g., $\alpha = .05 / 10 = .005$. **$p < .001$.
